# Establishment and characterization of a new spontaneously immortalized ER^−^/PR^−^/HER2^+^ human breast cancer cell line, DHSF-BR16

**DOI:** 10.1038/s41598-021-87362-0

**Published:** 2021-04-16

**Authors:** Stefania Nobili, Antonella Mannini, Astrid Parenti, Chiara Raggi, Andrea Lapucci, Giovanna Chiorino, Sara Paccosi, Paola Di Gennaro, Vania Vezzosi, Paolo Romagnoli, Tommaso Susini, Marcella Coronnello

**Affiliations:** 1 Department of Health Science, Section of Clinical Pharmacology and Oncology, University of Florence, viale Pieraccini, 6, 50139 Florence, Italy; 2 Department of Experimental and Clinical Medicine, University of Florence, Florence, Italy; 3 Fondazione Edo ed Elvo Tempia Valenta, Biella, Italy; 4 Plastic and Reconstructive Surgery Unit - Regional Melanoma Referral Center - Tuscan Tumor Institute (ITT), Santa Maria Annunziata Hospital, Bagno a Ripoli, Florence, Italy; 5 Department Organizational Structure (SOD) of Pathological Histology and Molecular Diagnostics, AOU Careggi, Florence, Italy; 6 Present Address: Department of Neurosciences, Imaging and Clinical Sciences, “G. d’Annunzio” University, Chieti, Italy

**Keywords:** Breast cancer, Cancer metabolism, Cancer models

## Abstract

Invasive ductal carcinoma (IDC) constitutes the most frequent malignant cancer endangering women’s health. In this study, a new spontaneously immortalized breast cancer cell line, DHSF-BR16 cells, was isolated from the primary IDC of a 74-years old female patient, treated with neoadjuvant chemotherapy and disease-free 5-years after adjuvant chemotherapy. Primary breast cancer tissue surgically removed was classified as ER^−^/PR^−^/HER2^+^, and the same phenotype was maintained by DHSF-BR16 cells. We examined DHSF-BR16 cell morphology and relevant biological and molecular markers, as well as their response to anticancer drugs commonly used for breast cancer treatment. MCF-7 cells were used for comparison purposes. The DHSF-BR16 cells showed the ability to form spheroids and migrate. Furthermore, DHSF-BR16 cells showed a mixed stemness phenotype (i.e. CD44^+^/CD24^−/low^), high levels of cytokeratin 7, moderate levels of cytokeratin 8 and 18, EpCAM and E-Cadh. Transcriptome analysis showed 2071 differentially expressed genes between DHSF-BR16 and MCF-7 cells (logFC > 2, *p*-adj < 0.01). Several genes were highly upregulated or downregulated in the new cell line (log2 scale fold change magnitude within − 9.6 to + 12.13). A spontaneous immortalization signature, mainly represented by extracellular exosomes-, plasma membrane- and endoplasmic reticulum membrane pathways (GO database) as well as by metabolic pathways (KEGG database) was observed in DHSF-BR16 cells. Also, these cells were more resistant to anthracyclines compared with MCF-7 cells. Overall, DHSF-BR16 cell line represents a relevant model useful to investigate cancer biology, to identify both novel prognostic and drug response predictive biomarkers as well as to assess new therapeutic strategies.

## Introduction

Breast cancer is the most common and lethal cancer in women worldwide^1^. Tumor and patient characteristics are classical factors that drive breast cancer therapy and predict prognosis together with the status of specific biomarkers, such as estrogen receptor (ER), progesterone receptor (PR) and human epidermal growth factor receptor 2 (HER2). These biomarkers contributed to the classification of five major subtypes of breast cancer: Luminal A (i.e. ER positive (ER^+^)/PR positive (PR^+^), HER2 negative (HER2^−^); Luminal B (i.e. ER^+^/PR^+^, HER2^+^ or HER2^−^ with high levels of Ki-67); Triple-negative/basal-like (i.e. ER^−^/PR^−^, HER2^−^); HER2-enriched (i.e. ER^−^/PR^−^, HER2^+^), and Normal-like (ER^+^/PR^+^, HER2^−^ with low levels of Ki-67), being the triple-negative subtype predictive off the worst prognosis.

However, breast cancer is a very heterogeneous disease as evidenced by genetic and gene expression profiling studies^2^ that further subclassify the above reported molecular subtypes. In particular, gene expression studies have further refined the ability to predict response to chemotherapy and prognosis by developing breast cancer genomic profiles (e.g. Oncotype DX) that can be used in selected cases^3^.

Overall, HER2 is amplified and overexpressed in 25–30% of human breast cancers. Although HER2^+^ breast cancer patients have a worse prognosis compared with HER2^−^ cancer patients, the availability of the anti-HER2 monoclonal antibody trastuzumab has changed the natural history of this tumor, thus significantly improving the overall survival of HER2^+^ breast cancer patients^4^. However, at least 85% of HER2^+^ overexpressing breast cancer is ER^−^ and PR^−^ at baseline^5^, thus preventing patients from undergoing hormonal therapy. On this basis, it is clear the need to identify new drugs as well as novel targets useful for the treatment of hormone receptor negative breast cancer.

Although it is well known the relevance of the complex interplay between cancer cells and tumor microenvironments in tumor response to treatment, in vitro studies based on established tumor cell lines still represent the starting point to design powerful in vivo and translational clinical studies^6^. Several established breast cancer cell lines, representative of the different mammary tumor subtypes^7^, are available and are commonly used to investigate breast cancer heterogeneity as well as breast cancer sensitivity to different drugs or to identify new potential pharmacological targets^8–10^.

Thus, the availability of additional appropriate cellular models may significantly improve the knowledge on breast cancer molecular heterogeneity, the discovery of new biomarkers useful in predicting drug response or to be used as drug targets.

Tumor established cell lines have often been obtained through immortalization protocols that may cause the selection of subpopulations with transformed phenotypes no longer representative of the original cancer tissue^11^. In this paper, we report the isolation and characterization of a spontaneously immortalized breast cancer cell line, a very rare phenomenon in primary breast cancer cultures, that shows human telomerase reverse transcriptase (*h-TERT*) expression levels similar to those of MCF-7 cell line, isolated in 1973^10^ by a pleural effusion of breast cancer, and that represents a classical in vitro model for the study of breast cancer features, including sensitivity/resistance to anticancer treatments.

Our breast cancer cell line, named DHSF-BR16 (i.e. Department of Health Science of Florence-Breast16), was derived from a patient affected by ER^−^/PR^−^, HER2^+^ primary IDC. We compared DHSF-BR16 with MCF-7 cells (ER^+^/PR^+^, HER2^−^) under identical culture conditions to highlight their molecular and functional differences in order to carefully characterize the biological properties of DHSF-BR16 cells and identify genes or pathways potentially relevant for new therapeutic strategies.

Thus, by establishing and characterizing this new cell line, our aim was to provide the scientific community with a new cellular model useful to further investigated human breast cancer.

## Materials and methods

### Patient information

The patient was a 74 years old female affected by locally advanced IDC. Prior to chemotherapy and subsequent surgery, the patient underwent needle biopsy. The histological diagnosis was infiltrating breast carcinoma with weak (< 1%) ER staining (clone SP1), negative for PgR (clone 1E2), 60% cells positive for Ki-67 (clone MIB1) and HER2 positive with intense staining > 10% (score 3+). Thus, the patient underwent neoadjuvant chemotherapy including liposomal doxorubicin and cyclophosphamide followed by paclitaxel and trastuzumab. Trastuzumab was also administered in the post-operative setting. A partial clinical and radiographic response was documented after neoadjuvant treatment with a 50% reduction of the tumor diameter. As a surgical procedure a quadrantectomy with sentinel node biopsy and removal of 2 additional I level axillary lymph nodes was performed. The histological and biological characteristics of the tumor were the following: high grade (G3) IDC not otherwise specified, ER^−^/PR^−^, HER2^+^ 40% (color intensity score 3+), 60% of cells positive for Ki-67 (i.e. highly proliferating tumor). The pathological stage evaluated post-neoadjuvant chemotherapy was: ypT1c; ypN0 (sn)(mol). The patient is disease-free 5-years after adjuvant chemotherapy. The patient gave her written informed consent to the use of part of the surgical specimen for the study purposes. The study was approved by the local ethics committee of the Careggi Hospital, University of Florence (Firenze, Italy), and all the relative procedures were performed in accordance with SOPs (Standard Operating Procedures) and relevant guidelines and regulations of the Careggi Hospital, University of Florence (Firenze, Italy).

### Tissue non-enzymatic processing

Tissue samples (primary tumor histologically confirmed to be not significantly contaminated by normal tissues, necrotic tissues and lymphocytes, and histologically normal tissue adjacent to the tumor), were rapidly frozen in liquid nitrogen. Within 30 min from surgery, a tumor tissue portion, was placed in Advanced DMEM/F12 medium with 5% fetal bovine serum (FBS) (Gibco), 2 mM glutamine and an antibiotic/antifungal solution (Gibco) containing 10,000 units/mL of penicillin, 10,000 µg/mL of streptomycin, and 25 µg/mL of amphotericin B, and immediately transferred to the laboratory for subsequent processing. Tumor tissue was manually separated from adipose areas using sterile scalpel and was minced into pieces < 1 mm^3^ with sterile scissors; the pieces were placed in a sterile microblade-equipped polyethylene chamber, medicon (Becton–Dickinson) with medium mentioned above. When the medicons were inserted into the Medimachine (Becton–Dickinson), the fragments came into contact with the blades and were dissociated from four to five times for 20 s at a constant speed < 100 r.p.m^12^. Medium containing recovered cells was filtered by porous polyester membranes (Becton–Dickinson). After filtration, cells were washed and transferred into a 1 mL plate and observed under an inverted microscope (Leitzs, Germany) in order to check the presence of organoids; subsequently organoids were separated from the other cellular fractions by centrifugation at 700 rpm for 2 min. Pellet, suspended in complete culture medium, was incubated at 37 °C in a humidified atmosphere at 5% CO_2_. After 24 h, organoids and single cells started the attachment process, and the supernatant, loaded in organoids and non-adherent cells, was transferred on a new plate. This operation was repeated every 48–72 h to get a cellular culture ever cleaner and to increase the number of available cellular samples. Differential trypsinization was used to get rid of the fibroblasts^13^. Briefly, cells were incubated with trypsin for a 1 min, until detachment of fibroblasts. Then, fibroblasts were discarded and the dish was washed 2–3 times with Advanced DMEM/F12 medium before adding fresh complete medium to the cells. Cultures were observed daily for cell growth and the medium was changed every three days (Supplementary Fig. S1).

### Establishment of the DHSF-BR16 cell line

DHSF-BR16 cell line, initiated from the primary IDC, took 24 months to establish in Advanced DMEM/F12 medium added with 3% FBS, 2 mM glutamine, and without antibiotic/antifungal solution. Cell subconfluence was achieved within 10 days and subculture ratio was 1:3/1:5. Cell aliquots were frozen under liquid nitrogen in different passages. Freezing medium was composed of Advanced DMEM/F12 medium/FBS/DMSO 40/50/10% (v/v). The recovery of cells and their adhesion to the flask after thawing were slow.

### Cytogenetic analysis

Growing cultures were harvested for karyotypic analysis. Optimal numbers of cells in mitosis were obtained by adding colchicine in a final concentration of 0.16 µg/mL of colture medium and incubated at 37 °C. After cell collection, the conventional protocol for chromosome preparation was applied^14^.

### Flow cytometry

All samples were acquired by a FACSCanto flow cytometer (Becton Dickinson) and for each sample 20,000 events were acquired and analyzed by FCS 6 Express software (De Novo Software).

#### DNA index

To evaluate the DNA index, unfixed DHSF-BR16 cells and human lymphocytes obtained from a health volunteer were stained with a Propidium Iodide (PI) staining technique^15^. The DNA index was calculated as the ratio of DHSF-BR16 cells and peripheral blood diploid (2n) lymphocytes G_0_/G_1_ fluorescence channels. According to Ross et al.^16^, a DNA index of 1.0 is indicative of diploidy whereas a ratio ranging from 0.85 to 1.9 is indicative of ipo- or hyper-diploidy, respectively.

#### Evaluation of duplication time and cell cycle progression

These growth parameters have been evaluated in the same experiment by cell counting and PI staining, respectively, according to the procedures described in Coronnello et al.^17^.

#### Expression of CD44/CD24 antigens

The expression of CD44 and CD24 surface antigens was measured after DHSF-BR16 and MCF-7 cell staining with CD44 and CD24 antibodies (Thermo Fisher), according to the manufacturer’s instructions (Supplementary Methods).

#### Detection of reactive oxygen species (ROS)

ROS production was evaluated using 2′,7′-dichlorodihydrofluorescein diacetate (DCFH-DA, Kodak)^18^. Fresh stock solution of 2 mM (w/v) DCFH-DA in ethanol was prepared and kept at − 20 °C in the dark until the use. Briefly, cells were incubated with DCFH-DA solution (10 µM) at 37 °C for 1 h in a humidified atmosphere with 5% CO_2_. After washing, the fluorescence of cells was acquired by FACS (i.e. λ_ex_ 488 nm, λ_em_ 530 nm).

### Electron microscopy

Cells were rinsed in saline (0.1 mol/L phosphate buffer, pH 7.4, 380 mOsm/L) and fixed with 2% formaldehyde and 2.5% glutaraldehyde in 0.1 mol/L cacodylate buffer, pH 7.4, osmicated and embedded in Epon. 1 µm-thick-sections, unstained or stained with alkaline toluidine blue, were observed by light microscopy, and about 70 nm-thick sections were observed by a JEM 1010 electron microscope (Jeol), at 80 kV, upon staining with gadolinium acetate followed by lead citrate or bismuth subnitrate^19^. The images were acquired by a digital camera MegaView III (Soft Imaging System GmbH) connected to a computer (Dell) equipped with a dedicated software (Soft Imaging System, AnalySIS 3.1 version; https://www.scientistlive.com/content/9182).

### Enzymatic and fluorescent immunocytochemistry

The analysis of ER, PR, and HER2 status was performed by an immunoenzymatic technique associated with immunofluorescence on a cellular spot obtained from the established cells (see details in Supplementary Methods). Cells were labeled with a large panel of antibodies (i.e. epithelial markers), including anti-ER/PR and anti-HER2 antibodies, and analyzed by a Leica DMLB light microscope equipped for epifluorescence (Leica Microsystems GmbH) for a qualitative analysis^20^.

### Fluorescent in situ hybridization (FISH)

The amplification of HER2 by FISH analysis was carried out according to ASCO/CAP HER-2 2018 guidelines^21^.

### Tumor spheroid-based migration on matrix protein

#### Generation of spheroids

The hanging drops technique according to Insphero (InSphero AG) manufacturer’s instructions was used. Single DHSF-BR16 and MCF-7 cells were seeded at various cell densities (ranging from 1000 to 8000) into special hanging drop culture plates. In order to obtain optimal spheroids (200–300 µm) without central necrotic area after 4 days of culture, an inoculum of 2000 cells/well was used. MCF-7 culture conditions are described in Supplementary Methods.

#### Migration method

Cell migration was studied by using tumor spheroids. 200–300 μm-spheroids were transferred into gelatin-coated flat-bottomed 96-well plates (a single spheroid/well) with addition of culture medium (T_0_). Cell migration was recorded every 24 h for a total period of 144 h. We obtained images using an inverted Leitz microscope (Leitz Corporation, Stuttgart, Germany), equipped with a 6MP Digital Camera (CCD vision sensor, square pixels of 4.4 µm side length, 1600 × 1200 pixel resolution, 8-bit grey level) (TiEsseLab, Italy). The ISCapture software (version 3.6.9.1; http://www.tiesselab.com) was used to obtain 2D morphological parameters (diameter, perimeter, area) as well as to select morphologically homogeneous spheroids and to measure degree of the migration in the time^22^.

### Gelatinase activity by zymography

Gelatinase MMP2 and MMP9 activity, involved in matrix remodeling and tumor cell invasion, was measured as reported in Paccosi^23^, by quantitative densitometry (see Supplementary Methods).

### RNA preparation

Total RNA was extracted from primary tumor and normal tissues and from DHSF-BR16 and MCF-7 cells with the RNeasy kit (Qiagen) according to the manufacturer’s instructions. The RNA concentration and quality were measured using an optical NanoDrop ND1000 spectrophotometer (Thermo Scientific). The RNA integrity was assessed by the Agilent 2100 Bioanalyzer (Agilent Technologies Inc.), according to the product user guide. All RNA samples used in this study had the 260/280 ratio > 1.9 and an RNA Integrity Number (RIN) from 5 to 9.

### Quantitative real-time PCR (RT-qPCR)

Total RNA (500 ng) from primary tumor and normal tissues and from DHSF-BR16 and MCF-7 cells were transcribed to cDNA with a High-Capacity cDNA Reverse Transcription Kit (Applied Biosystems).

Changes in the mRNA expression level of 38 candidate genes selected on the basis of their biological role in cancer, were detected using FAST SYBR-Green PCR Master Mix and the ABI ViiA 7 System (Applied Biosystems). Ribosomal 18S RNA (rRNA) and glyceraldehyde 3-phosphate dehydrogenase (GAPDH) were used as a normalizer for *h-TERT* and all the other candidate genes, respectively.

The fold difference (2^−ΔΔCt^) was calculated using the ΔCt of MCF-7 as control. All reactions were performed in triplicate. Primers used for RT-qPCR are listed in Supplementary Table S1.

### Microarray expression profiling

Transcriptome analysis was performed on RNA samples of DHSF-BR16 and MCF-7 cells obtained from three independent pellets as reported above .

Gene expression profiling was carried out using the one-color labelling method: labelling, hybridization, slide washing and scanning were performed following the manufacturers protocols (Agilent Technologies). Briefly, mRNA from 100 ng of total RNA was amplified, labeled with Cy3 and purified with columns. Labeled samples (600 ng) were hybridized on Agilent Human Gene Expression 8 × 60 K v3 microarrays. After 17 h, slides were washed and scanned using the Agilent Scanner version C (G2505C, Agilent Technologies). The fluorescence intensities of scanned images were extracted and pre-processed by Agilent Feature Extraction Software (v10.7.3.1).

#### Bioinformatic analysis

Differential expression analysis was performed by using the R Bioconductor repository (http://www.bioconductor.org) limma package. Raw intensities were background corrected and normalized with the quantile function. Signals from replicate probes were averaged. Differentially expressed genes (DEGs) were retrieved by combining linear models with empirical Bayes analysis and modified *t* test raw *p* values were adjusted for multiple testing by using Benjamini–Hochberg procedure.

#### Functional analysis

DAVID software (Database for Annotation, Visualization and Integration Discovery; http://david.abcc.ncifcrf.gov/) for functional enrichment analysis of the DEGs in DHSF-BR16 cells compared with MCF-7 cells was used. Over-represented Biological Processes, Cellular Components and Molecular Functions of the Gene Ontology (GO) database, as well as KEGG pathways were retrieved by setting to 0.01 the threshold of the enrichment *p*-value.

### Drug sensitivity assay

The best cell inoculum density for the determination of doxorubicin sensitivity was preliminarily defined by the sulforhodamine B (SRB) assay^17^. Briefly, DHSF-BR16 cells were seeded onto 96-well microplates (Sarstedt) at a density of 1.0, 1.5, 2.0, 3.0 × 10^4 ^cells/well. After 24 h of incubation at 37 °C in a humidified atmosphere with 5% CO_2_, the culture medium was replaced with fresh medium and doxorubicin was added at final drug concentrations varying from 3 × 10^–8^ to 1 × 10^–4^ M. After 72 h of incubation, the assay was terminated by the addition of cold TCA and the cells were stained according to the procedure reported in Coronnello et al.^17^ to determine the IC_50_ values. After choosing the optimal cell density, the sensitivity of DHSF-BR16 and MCF-7 cells (same cell density/well) to epirubicin, docetaxel and paclitaxel was evaluated at pharmacological concentrations ranging from 1 nM to 10 µM for epirubicin and from 0.003 nM to 0.1 µM for taxanes. The pharmacological sensitivity of the study cell line was compared with that of the MCF-7 reference line and was indicated by the resistance index (RI) obtained from the ratio between the IC_50_ values of the two cell lines for each individual drug. Respect to MCF-7 line, RI values higher than 1 indicate resistance of DHSF-BR16 cells to the tested drug and values lower than 1 indicate drug sensitivity. Cytotoxicity data are represented as mean ± standard error (SE).

### Statistical analysis

Student *t*-test was used for pairwise comparison. Statistical analysis was performed by GraphPad version 5 (Graph Pad Prisme Software Inc., La Jolla, CA) or as specified in the previous paragraphs, *p* values < 0.05 were considered statistically significant.

## Results

### *h-TERT* expression, cytogenetic, morphologic and growth characteristics of DHSF-BR16 cells

**Figure 1:**
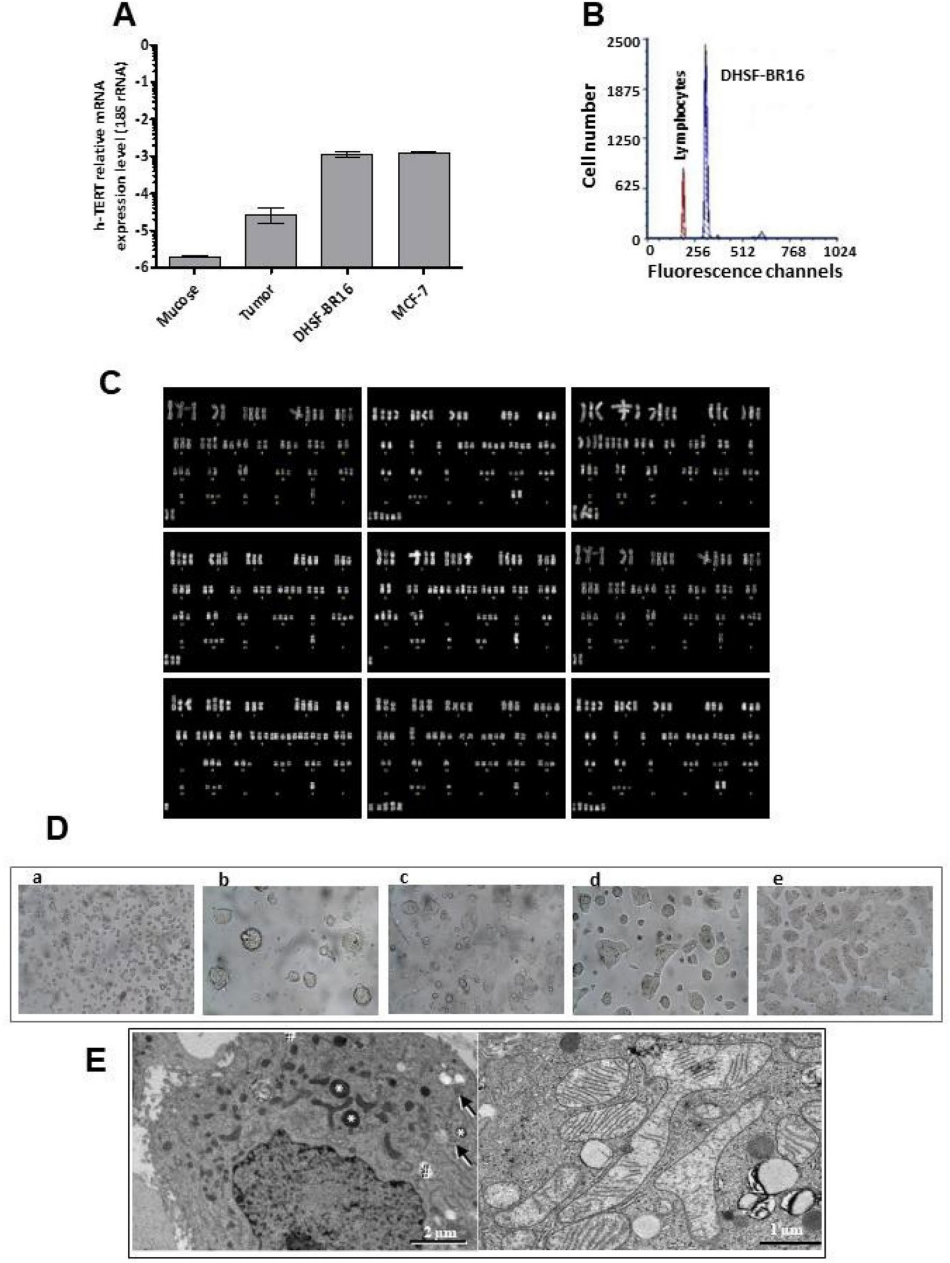
*h-TERT* gene expression, cytogenetic, morphologic and growth characteristics of DHSF-BR16 cells. (**A**) mRNA expression levels of *h-TERT* in DHSF-BR16 and MCF-7 cells, breast cancer tissue and the corresponding normal tissue. (**B**) Aneuploidy of DHSF-BR16 cells by the DNA analysis. DHSF-BR16 and human peripheral blood lymphocytes G_0_/G_1_ fluorescence channel is 318 and 195, respectively. (**C**) Representative karyotype from DHSF-BR16 cell line with different number of chromosomes, various chromosomal rearrangements and unidentifiable marker chromosomes. Chromosome analysis was performed at 10th and 30th passages with similar findings. (**D**) First subculture of DHSF-BR16 established cell line: (a) cells splitted; (b) 1 day from split; (c) 4 days from split; (d) 7 days from split; (e) 10 days from split, showing patchy appearance. × 40 magnification (a), × 200 magnification (b–e). (**E**) DHSF-BR16 cell morphology by electron microscopy: left, isolated cell, with short, sparse microvilli on the cell surface. Asterisks: electron dense lysosomes; hashtags: lipid droplets; arrows: arrays of glycogen particles; right, mitochondria round or elongated up, occasionally branched. There were very large mitochondria, elongated and more than 3 µm long and with irregular dilations up to 2 µm, with variable amount of cristae.

As a first approach, we analyzed the expression of the *h-TERT* gene, a component of the immortalization signature by RT-qPCR. Results showed low or lack of *h-TERT* expression levels in the primary tumor and in normal tissue, respectively. Conversely, high levels of *h-TERT* mRNA were observed in DHSF-BR16 and, as expected, in MCF-7 cells that are known to endogenously express this gene (Fig. 1A).

FACS analysis showed that DHSF-BR16 cells had a DNA index of 1.6, thus indicating a relevant percentage of aneuploid tumor cells (Fig. 1B), in agreement with cytogenetic findings (Fig. 1C).

The modal chromosome number of the DHSF-BR16 cell line was 56, with a range of 49–63 chromosomes/cell. The DHSF-BR16 karyotype (Fig. 1C) was characterized by numeric alterations and the presence of unidentifiable marker chromosomes. Several chromosomes were absent, and others were clearly under- or over-represented.

The DHSF-BR16 cell line displayed an epithelial morphology with patchy appearance, as compact multi-layered colonies (Fig. 1D). DHSF-BR16 cells rarely became confluent and many materials, potentially of secretory origin, and some cellular debris were present in the culture supernatant. DHSF-BR16 cells were required to be split every 10 days: a longer period implicated a strong adhesion to the flask, making also more difficult the detachment (Fig. 1D, e). After 24 h seeding (Fig. 1D, b), many spontaneous spheroids and viable cells were in suspension, and the percentage of adherent cells was 25%.

The morphology of DHSF-BR16 isolated cells was studied by electron microscopy (Fig. 1E). Isolated cells were 10–15 µm in diameter, had a round or oval nucleus often with shallow indentations, loose chromatin and large nucleolus. The cell surface expanded into many short microvilli. Mitochondria were round up to 300 nm wide or elongated up to ~ 1 µm, occasionally branched. There were occasional very large mitochondria, with a variable amount of cristae.

The DHSF-BR16 cells progression through the cell cycle phases as a function of the time was studied (Supplementary Fig. S2). Interestingly, the highest percentage of cells under division (G_2_M 13.7%) was reached within 48 h; in the later times, the slowdown in proliferation was confirmed by a moderate and constant mitotic activity which ensured a constant proliferative potential. The duplication time was evaluated as reported in Supplementary Fig. S2.

### Remarkable ROS generation in DHSF-BR16 cells

**Figure 2:**
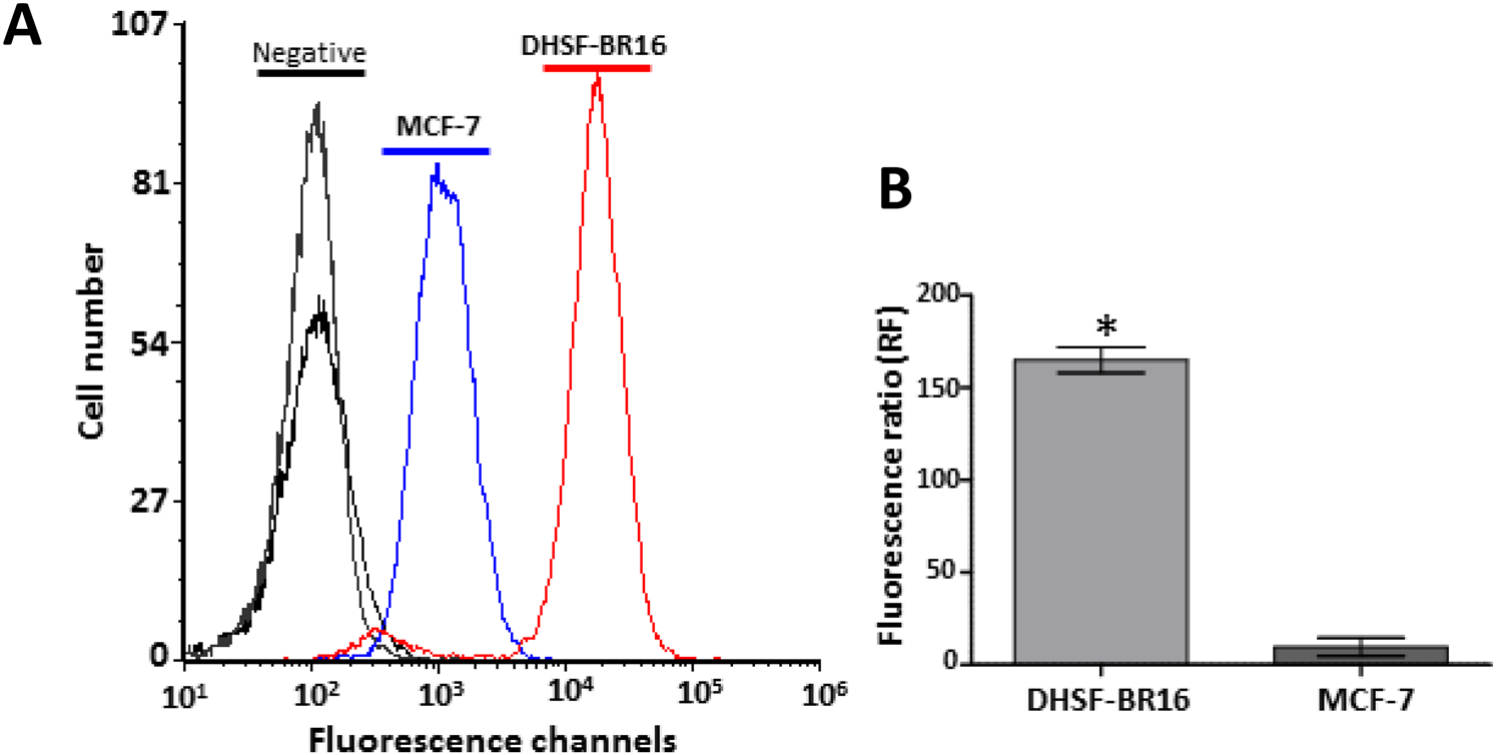
ROS generation in DHSF-BR16 and MCF-7 cells. In the presence of ROS, DCFH-DA is converted to a fluorescent compound. (**A**) Fluorescence curves are representative of three experiments. (**B**) Fluorescence Ratio between mean fluorescence intensity (MFI) of DCFH-DA-treated cells and negative samples. The results are presented as mean ± SE of three replicates. *p < 0.0001.

Due to the fundamental role of ROS in cancer initiation, development and progression, the basal oxidative stress of DHSF-BR16 cells was evaluated by FACS analysis and compared with that of MCF-7 cells (Fig. 2). The ROS generation was about 17-fold higher in DHSF-BR16 than in MCF-7 cells, as shown by the Fluorescence Ratio (FR) value (FR 165 and 9.6, respectively).

### DHSF-BR16 cell molecular setting and biomarker characterization

**Figure 3:**
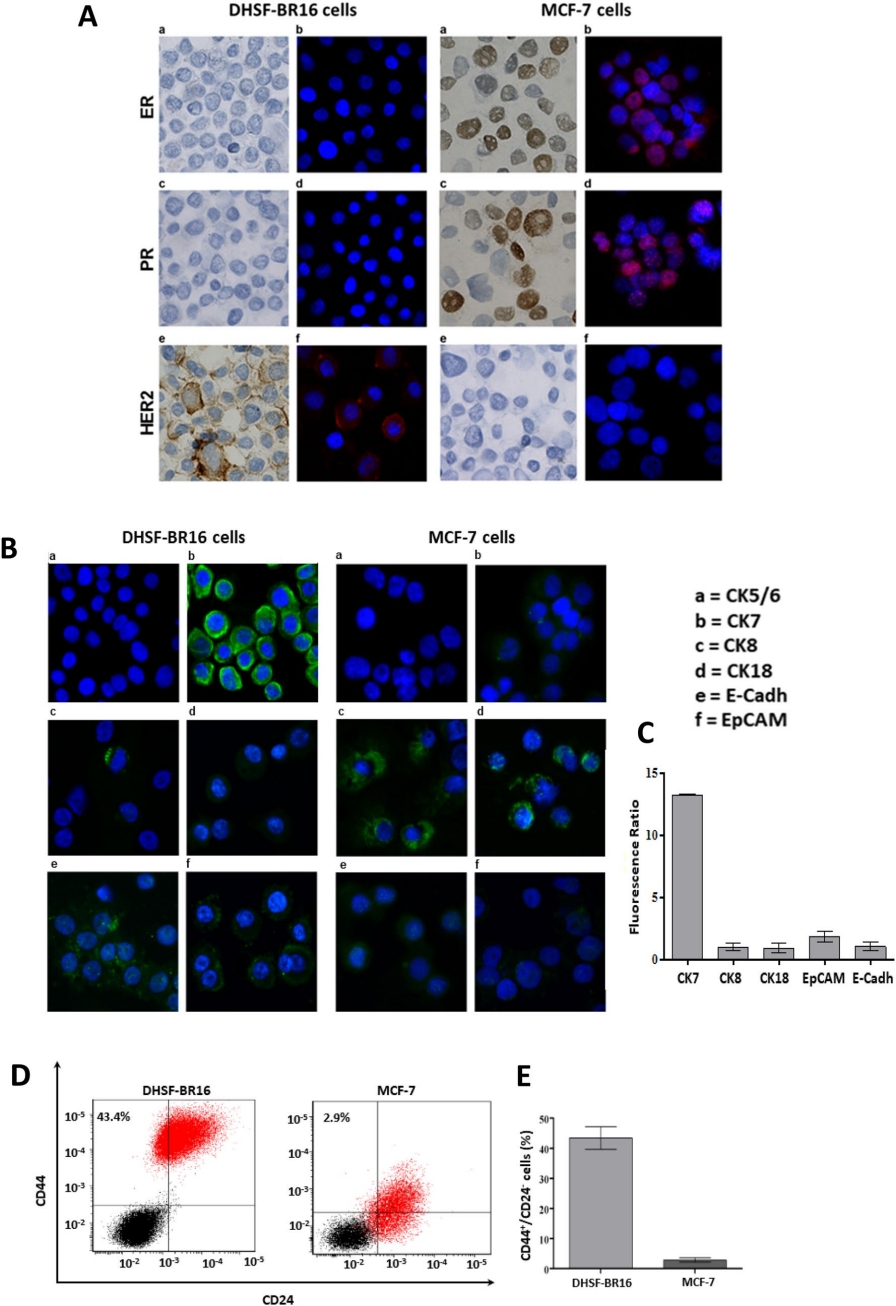
Fluorescent immunocytochemistry and FACS analysis of cell-surface markers, CD44 and CD24 in DHSF-BR16 and MCF-7 cells. (**A**) Immunocytoenzymatic (a,c,e) and immunocytofluorescence (b,d,f) analyses of ER, PR, and HER2 receptors expression in DHSF-BR16 and MCF-7 cells, respectively. Analyses were performed on cytospins with the following anti-human Abs: estrogen receptor (ER) (rabbit, clone SP1); progesterone receptor (PR) (rabbit, clone 1E2); HER2 (rabbit, clone 4B5) (original × 400 magnification); (**B**) Analyses were performed on cytospins fixed in cold acetone and with the following anti-human Abs: CK7 (rabbit, clone SP52); CK5/6 (mouse, clone D5/16B4). Other primary human Abs tested are: CK8 (mouse, clone N1C1), CK18 (mouse, clone N2C2), EpCAM (mouse, clone N3C3) and E-Cadh. Nuclei were labeled with DAPI nuclear stain. (original 400 × magnification); (**C**) the immunofluorescence signal quantification was performed using FIJI software; for each antibody, the fluorescence ratio based on the DHSF-BR16 and MCF-7 fluorescence value was calculated. (**D**) Flow cytometric representative plots of receptor profile (CD44/CD24) expression measured in DHSF-BR16 and MCF-7 cultured in adherent conditions. The percentages refer to the sample stained with Abs (red) and overlayed on the negative sample (black); (**E**) proportion of cancer stem cells with CD44^+^/CD24^−^ profile; results are the means ± SD of at least 3 experiments. An anti-CD24 (PE conjugate, clone SN3) and an anti-CD44 (APC conjugate, clone IM7) were used.

To evaluate the long-term maintenance of the molecular setting (i.e. ER^−^/PR^−^/HER2^+^) of the primary tumor, the immunoenzymatic and immunofluorescent analyses were repeated on a cellular spot obtained from DHSF-BR16 cells kept in culture for about 2 years, confirming the ER^−^/PR^−^/HER2^+^ phenotype in DHSF-BR16 cells, and the ER^+^/PR^+^/HER2^−^ phenotype, belonging to MCF-7 cells (Fig. 3A). Also, the amplification of HER2 has been further evaluated by FISH analysis (Figure S3), and the HER2 positive phenotype of DHSF-BR16 cells as well as the HER2 negative phenotype of MCF-7 cells has been confirmed.

DHSF-BR16 and MCF-7 cells were also characterized for cytokeratins (CKs) (i.e. CK5/6, CK7, CK8 and 18), EpCAM and E-Cadh expression by fluorescent immunocytochemistry (Fig. 3B). Compared to MCF-7 cells, the DHSF-BR16 cells showed a strong expression of CK7 (13 times), a low expression of CK8 and CK18 (< 1), and lack of expression of CK5/6, in both cell lines (data not shown). Finally, E-Cadh and EpCAM fluorescence in DHSF-BR16 cells was moderate, although higher than that reported in MCF-7, (i.e. 1.4 and 2.3 fold increase, respectively) (Fig. 3C).

Concerning the expression of CD44/CD24 cell-surface markers (Fig. 3D), FACS analysis revealed 43.4% of CD44^+^/CD24^−^ cells in the DHSF-BR16 cell line (56.6% of CD44^+^/CD24^low^). In contrast, MCF-7 cells showed 2.9% of CD44^+^ /CD24^−^ cells (46.7% of CD44^+^/CD24^low^). Also, DHSF-BR16 cells displayed a higher fluorescence intensity of CD44 compared to MCF-7 cells.

### Migration and invasivity

**Figure 4:**
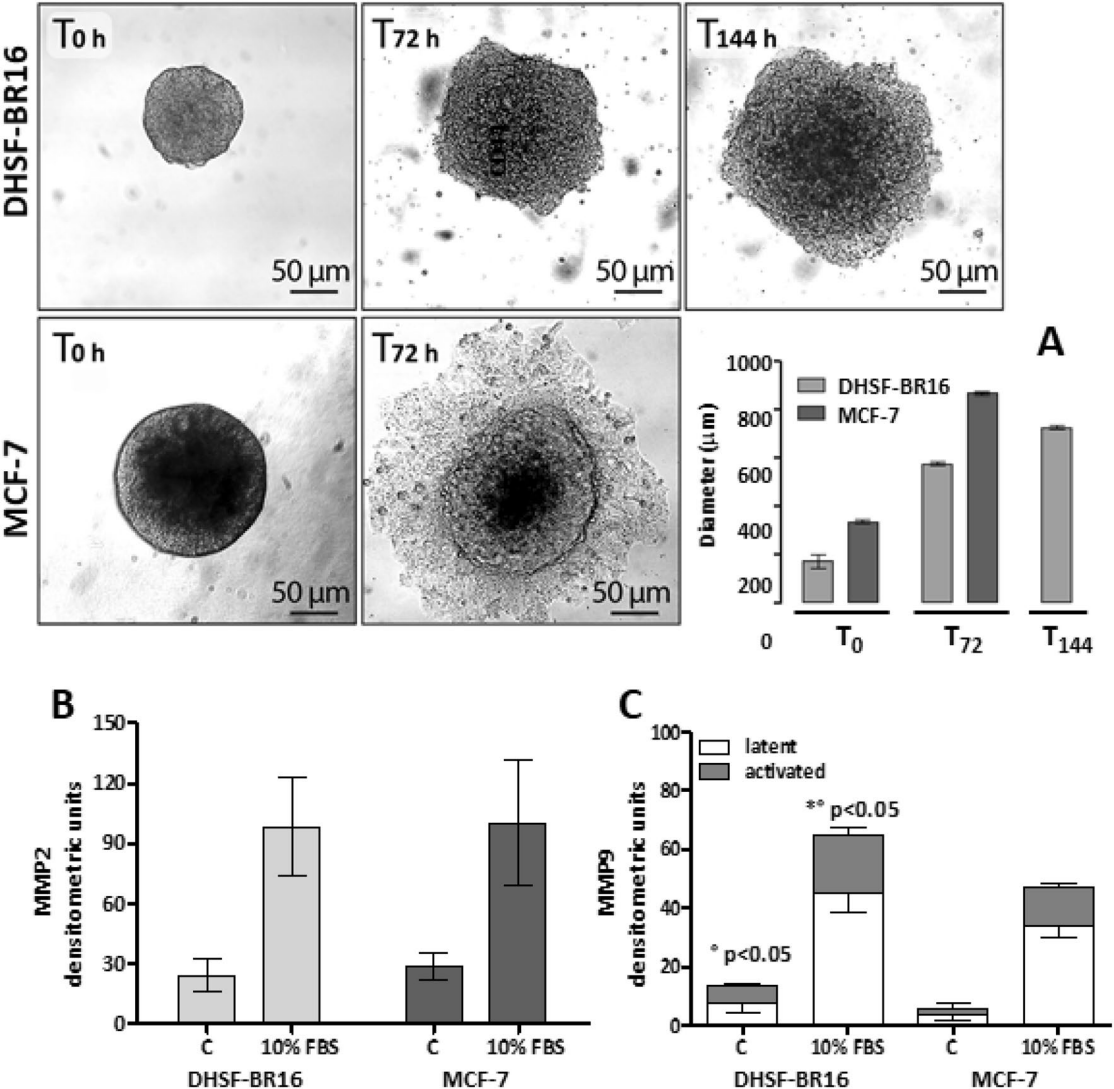
Migration and MMPs activities. Representative brightfield images of DHSF-BR16 and MCF-7 spheroid migration process, at T_0_, T_72_ and T_144_ h (intermediate time images not shown). (**A**) The graph shows the evaluation means of the spheroid diameters at the selected times. Data are the mean ± ES of at least three measurements. Images were obtained by using an inverted Leitz microscope , equipped with a 6MP Digital camera (CCD vision sensor, square pixels of 4.4 μm side length, 1600 × 1200 pixel resolution, 8-bit grey level) ). In (**B**) (MMP2) and (**C**) (MMP9), isoforms under basal conditions and FBS stimulation.

200–300 µm spheroids were transferred to gelatin (1:100) coated plates. In Fig. 4, we reported representative images of DHSF-BR16 and MCF-7 spheroid migration processes, and the graph (Fig. 4A) with the evaluation means of the spheroid diameters, measured at various times of the migration test. DHSF-BR16 migrating cells exhibited a very compact and multi-layered migration pattern, reaching a diameter comparable to that of MCF-7 cells (about 800 µm) at 144 h. MCF-7 spheroid cell dissemination was rapid with a dispersed and monolayered migration pattern.

MMP2 and MMP9 activities have been compared in DHSF-BR16 and MCF-7 cells. Gelatin zymography of both cell lines supernatants showed a constitutive release of the latent isoform of MMP2 (band at 72 kDa), which increased following 10% serum stimulation (Fig. 4B). No statistical differences in MMP2 activity were observed. The latent MMP9 isoform (92 kDa) was detected in both cell lines, although at a lesser extent, which increased after serum stimulation (Fig. 4C). Interestingly, both the latent and the activated MMP9 isoforms of DHSF-BR16 were significantly increased (*p* < 0.05) compared to those of MCF-7 cells.

### Transcriptome analysis

**Figure 5:**
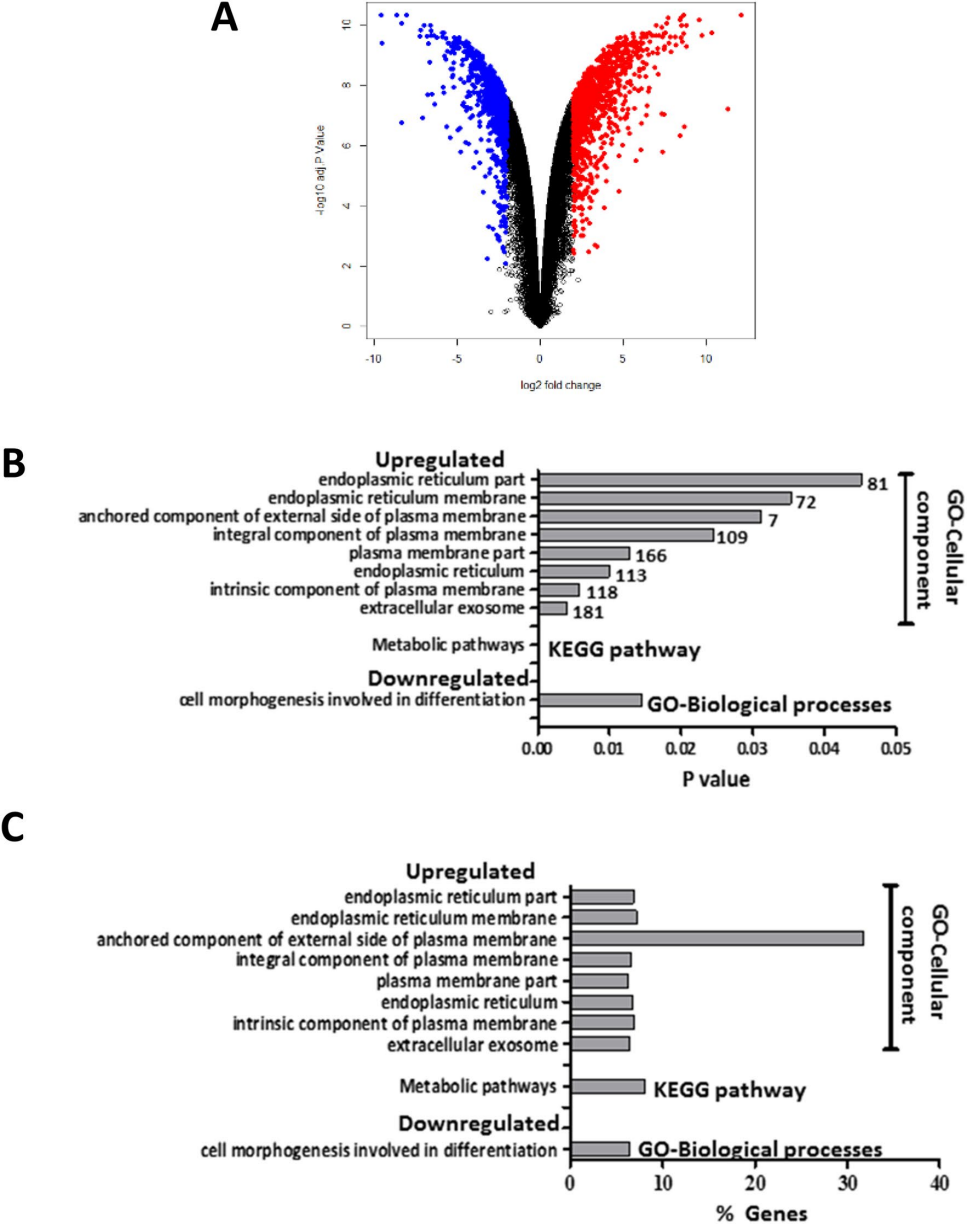
Volcano-plot of differentially expressed genes (DEGs) between DHSF-BR16 and MCF-7 cells. (**A**) DEGs were retrieved by combining linear models with empirical Bayes analysis. Modified *t* test raw *p* values were adjusted for multiple testing by using Benjamini–Hochberg procedure. Red dots and blue dots represent upregulated and downregulated DEGs, respectively. Black dots, no differential expression. Based on logFC > 2 and *p*-adj < 0.01, a total of 1214 upregulated and 857 downregulated genes were identified. The magnitude of fold changes (log2 scale) was within − 9.6 to + 12.13. (**B**) Number of genes included in the significantly upregulated or downregulated pathways, based on the GO and KEGG enrichment analysis. Grey bars indicate the magnitude of statistical significance. Numbers at the end of grey bars represent the number of genes for each pathway. (**C**) Percentages of genes included in the significantly upregulated or downregulated enriched pathways on the total of genes included in each pathway.

A total of 58,201 genes were successfully evaluated in both cell lines. Overall, 2071 (3.6%) out of 58,201 genes were differentially expressed (logFC > 2, *p*-adj < 0.01) in DHSF-BR16 cells compared with MCF-7 cells (Supplementary Table S2). In particular, 1214 genes were upregulated and 857 genes downregulated in DHSF-BR16 cells compared with MCF-7 cells. The magnitude of fold changes (in log2 scale) was within − 9.6 to + 12.13 (Fig. 5). Interestingly, among DEGs, a relevant number of genes involved in oxidative metabolism (e.g. *GSTP1*, *CYP4Z1, AKR1B10)* and in different pathways affecting breast cancer behavior (*WNT5A*), was observed.

### Functional enrichment analysis of DEGs

DEGs between DHSF-BR16 and MCF-7 cells were subjected to KEGG pathways and GO terms functional enrichment (Supplementary Table S2, Fig. 5B,C). KEGG pathway analysis revealed metabolic pathway (hsa: 01100) as the most significant pathway among genes upregulated in DHSF-BR16 cells whereas no pathway with significant functional terms was observed among downregulated DHSF-BR16 genes. GO analyses of the DEGs showed that cell morphogenesis involved in differentiation (GO:0000904) was the most enriched biological process term overrepresented among genes downregulated in DHSF-BR16. Among genes upregulated in DHSF-BR16, no enriched biological process term reached statistical significance. Among genes upregulated in DHSF-BR16, extracellular exosome (GO:0070062) was the most enriched term under cellular components followed by intrinsic component of plasma membrane (GO:0031226), plasma membrane part (GO:0044459), endoplasmic reticulum (GO:0005783), integral component of plasma membrane (GO:0005887), endoplasmic reticulum membrane (GO:0005789), anchored component of external side of plasma membrane (GO:0031362), endoplasmic reticulum part (GO:0044432). Among genes downregulated in DHSF-BR16, no enriched cellular component term reached statistical significance. Finally, no molecular function term reached statistical significance either among down- or up-regulated genes in DHSF-BR16.

### Quantitative real-time PCR of candidate genes

Among 38 candidate genes, selected according to their biological role in cancer initiation and progression, a total 14 genes were found to be differentially expressed between DHSF-BR16 and MCF-7 cell lines (Table 1, Supplementary Fig. S4). In particular, 12 genes were significantly up-regulated in DHSF-BR16 cells compared with MCF-7 cells, and 2 down-regulated. Up-regulated genes were *CD133* (*p* < 0.0001) that belong to the embryonic stem cell proliferation pathway, *EpCAM* (*p* < 0.0001), *CD44* (*p* = 0.0362) and *CLDN3* (*p* < 0.0001) that belong to the cell–cell adhesion and junction pathways, *SOX2* (*p* < 0.0001), *YAP1* (*p* < 0.0001), *STAT3* (*p* < 0.0001), and *HIF2α* (*p* < 0.0001) that belong to the transcriptional regulatory pathway, *CD24* involved in the pathway of modulation-growing and differentiation of B cells (*p* < 0.0001), *VEGFA* that is associated to the epithelial-mesenchymal transition (EMT) pathway (*p* = 0.0034), *WNT5A* that belongs to the differentiation pathway (*p* = 0.0002), and *ABCB1* that belongs to the ABC transporter pathway (*p* = 0.0006). *CLDN4* and *CLDN7* both involved in the cell–cell adhesion and junction pathways were instead found to be down-regulated in DHSF-BR16 cells (*p* < 0.0001 for both genes).

**Table 1:** Significant fold change variation of mRNA expression of candidate genes between DHSF-BR16 cells and MCF-7 cells.

Gene name	Description	Fold change^a^
**Up-regulated genes**		
*HIF2α*	Endothelial PAS domain protein 1	9.3 × 10^2^
*CD133*	Prominin 1	3.8 × 10^2^
*CD44*	CD44 molecule	39.3
*WNT5A*	Wnt family member 5A	10.2
*VEGFA*	Vascular Endothelial Growth Factor A	6.5
*YAP1*	Yes1 Associated Transcriptional Regulator	3.1
*STAT3*	Signal Transducer and Activator of Transcription 3	2.9
*ABCB1*	ATP binding cassette subfamily B member 1	2.8
*CLDN3*	Claudin 3	2.4
*CD24*	CD24 Molecule	1.9
*SOX2*	SRY-Box Transcription Tactor 2	1.6
*EPCAM*	Epithelial Cell Adhesion Molecule	1.2
**Down-regulated genes**		
*CLDN4*	Claudin 4	0.5
*CLDN7*	Claudin 7	5.0 × 10^–4^

Data obtained by RT-qPCR analysis.

^a^*t* test, *p* < 0.05.

### Sensitivity to anticancer drugs

Since the primary tumor underwent standard neoadjuvant chemotherapy based on anthracyclines and taxanes, sensitivity/resistance of DHSF-BR16 cells to a panel of cytotoxic drugs commonly used in the treatment of breast cancer (i.e. doxorubicin, epirubicin, paclitaxel and docetaxel) was evaluated and compared with that of MCF-7 cells.

Preliminary tests with different cell densities indicated a variability in IC_50_ values when cell density exceeds 15,000 cells/well, and therefore this latter cell density was selected to perform cytotoxicity studies (Supplementary Fig. S5). Cytotoxicity curves are shown in Supplementary Fig. S6.

Results reported in Table 2 show that both cell lines are more sensitive to epirubicin than doxorubicin, although DHSF-BR16 cells were more resistant to doxorubicin and epirubicin, compared with MCF-7 (RI values 3.8 and 1.5, respectively). Both cell lines were highly sensitive to taxanes mainly to paclitaxel although DHSF-BR16 cells were more sensitive than MCF-7 cells (RI 0.34 for docetaxel and 0.31 for paclitaxel).

**Table 2:** IC_50_ values of anticancer drugs tested on DHSF-BR16 cell line compared to the breast cancer cell line MCF-7.

Drugs	Cell lines (IC_50_ nM)^a^		
	DHSF-BR16	MCF7	RI
Doxorubicin	490 ± 120	130 ± 1.7	3.80
	*p* < 0.01		
Epirubicin	34 ± 5.6	23 ± 1.2	1.50
	NS		
Docetaxel	2.0 ± 0.2	5.9 ± 1.2	0.34
	*p* < 0.01		
Paclitaxel	0.16 ± 0.018	0.52 ± 0.014	0.31
	*p* < 0.01		

^a^The IC_50_ values are the mean ± ES of at least three experiments conducted in quadruplicate. *p* calculated as *t *test; NS, Not Significant.

## Discussion

We reported the establishment and characterization of a novel breast cancer cell line, named DHSF-BR16, derived from an ER^−^/PR^−^/HER2^+^ primary invasive ductal breast carcinoma of a female patient, still alive and disease-free 5 years from adjuvant chemotherapy. The above clinical molecular profile was maintained in the developed tumor cell line.

We were particularly interested in investigating the biological characteristics of the developed cell line. For this purpose and supported by highly informative studies^24,25^, we used the well known in vitro model MCF-7 as comparator. In fact, we exploited the opposite phenotypic setting of MCF-7 cells compared with the DHSF-BR16 cells to highlight the biological and molecular differences between the two tumor cell lines.

Results showed that the main phenotypic characteristic of this tumor model was the spontaneous cell immortalization, together with other relevant features such as the observed high oxidative stress, and the stemness profile. Furthermore, we examined the sensitivity/resistance of DHSF-BR16 cells to anticancer drugs and the gene-expression pattern by transcriptome analysis, to better highlight some crucial molecular features of this new in vitro tumor model.

Cell immortalization is a long-standing recognized hallmark of cancer^26^. The spontaneous immortalization is a quite rare event since primary cultures, after a few duplication cycles, commonly lose their replicative capability. In agreement with telomerase reactivation, an important prerequisite for achieving immortalization, we found a higher expression of *h-TERT* mRNA levels in DHSF-BR16 cells compared to primary tumor and normal tissues. Interestingly, the expression of *h-TERT* in DHSF-BR16 cells was comparable to that of the stabilized MCF-7 cells.

Telomerase enzyme can be partially responsible for the observed cancer cell survival, due to its important role not only in telomere elongation but also in functions related to key genes involved in proliferation, apoptosis, DNA damage response^27^, and differentiation^28^.

The DHSF-BR16 spontaneous immortalization signature was mainly represented by genes of the extracellular exosomes pathway, plasma membrane and endoplasmic reticulum membrane pathways, according to the GO database as well as by those related to metabolic pathways according to the KEGG database.

A peculiar feature observed in DHSF-BR16 cells was represented by a very high basal oxidative stress, despite a low cell death. In fact, the high levels of oxidative stress in DHSF-BR16 cell line did not confer a high sensitivity to anthracyclines compared with the MCF-7 cell line, that did not display the same ROS levels. We can hypothesize that the high level of oxidative stress observed in DHSF-BR16 cells may be partially due to the neoadjuvant treatment received by the patient, based on anthracyclines and trastuzumab^29^: such treatment could have selected tumor cells resistant to the oxidative stress. In addition, based on results of Indran et al.^30^, the *h-TERT* overexpression could have potentiated the anti-oxidant defense systems, enabling cells to elude death stimuli. The remarkable ROS production in DHSF-BR16 cells could be related to the presence of many elongated mitochondria, sometimes very large, detected by electron microscope examination. Furthermore, since the overexpression of *h-TERT* and the oxidative stress increase cancer stem cell subpopulation (CSC)^31^, and CSC subpopulation is identified by CD133^32^ and CD44^+^/CD24^−^ phenotype, it is notable that DHSF-BR16 cells showed an up-regulation of CD133 and a CD44^+^/CD24^−/low^ mixed phenotype, confirming the stemness profile.

In addition, from a pathophysiological point of view it is accepted that ROS impact the cellular energy metabolism by regulating key metabolic enzymes as well as metabolic pathways (e.g. glycolysis, pentose phosphate pathway, tricarboxylic acid cycle)^33^.

In keeping with this, our functional enrichment analysis of DEGs between DHSF-BR16 and MCF-7 cells evidenced a statistically significant difference related to genes involved in metabolic pathways (KEGG pathway analysis). In particular, genes involved in metabolic pathways were upregulated in DHSF-BR16 cells compared with MCF-7 cells.

Overall, these findings suggest that favorable in vitro growth conditions contributed to activate the phenotype of spontaneous immortalization.

Recently, in MCF-7 and MDA-MB231 cells, it has been shown that CD44 expression is closely related to the hypoxia-inducible factor-2α (HIF-2α), suggesting an important role of HIF-2α in maintaining stemness^34^. This transcript is highly upregulated in our cell model, according to both transcriptome and RT-qPCR analyses. Interestingly, Bai et al.^34^ also investigated the effects of HIF-2α silencing on the formation of mammospheres in MDA-MB231 cells, confirming its role in the efficiency of mammosphere formation. These data contribute to further highlight the stem traits of DHSF-BR16 cells, due to their ability to form mammospheres and to migrate, although through a different fashion, from MCF-7 cells. The non-radial and multi-layered modality of migration could be accounted for by signaling pathways that regulate various forms of movement, involved in tumor metastasis^35^. The invasion test results in DHSF-BR16 cells showed that latent and activated MMP9 isoforms were higher than those of MCF-7 cells. MMP9 has been demonstrated to correlate with more aggressive subtypes of breast cancer^36^ and to modulate metastatic cascade and immune response in the same tumor^37^. These findings suggest, as expected from a theoretical point of view, that the phenotype of the developed tumor cell model might be more aggressive than MCF-7 cells. However, these aspects warrant further investigation.

The immunofluorescence analysis highlighted the absence of CK5/6, thus further confirming that new cell model does not represent the basal-subtype, whereas the high expression of CK7 has been associated with HER2^+^^38^. As far as CK8 and CK18 expression is concerned, it has been demonstrated that the lack of CK8 and CK18 expression is responsible for highly invasive and dedifferentiated phenotype^39^. Therefore, our cell model, showing a very low expression of CK8 and CK18 compared to MCF-7 cells, could gain a more aggressive phenotype.

Another marker of notable value in the breast cancer prognosis is EpCAM, although literature data are still controversial^40^. In order to better understand the correlation HER2^+^/EpCAM, the HER2^+^ breast cancer DHSF-BR16 cells endowed by a moderate expression of EpCAM, may represent a suitable in vitro tumor model.

Transcriptome analysis identified 2071 DEGs between DHSF-BR16 and MCF-7 cells. Among such genes, in consideration of the above discussed DHSF-BR16 cell characteristics, we focused our attention on a few of them, among the upregulated ones, in order to provide some potential suggestions for exploiting this new tumor model. In particular, we referred to *GSTP1* and *AKR1B10* (GO:0070062 extracellular exosome), *CYPAZ1* and *WNT5A* (GO:0005783 endoplasmic reticulum), that are involved in relevant metabolic or detoxification pathways.

Although the role of *GSTP1* in predicting prognosis and chemoresistance in breast cancer patients has long been recognized, more recently high levels of *GSTP1* have been reported in exosomes of patients treated with neoadjuvant chemotherapy based on anthracyclines and taxanes^41^. Interestingly, the levels of *GSTP1* in exosomes from patients with progressive or stable disease were significantly higher than those in patients who obtained an objective response. Thus, *GSTP1*-containing exosomes could represent a potential biomarker predictive of response to standard neoadjuvant chemotherapy, in addition to other upregulated genes in exosomes^42^.

As known, the P450 cytochrome protein CYP4Z1 is preferentially expressed in breast cancer^43^. Furthermore, increased CYP4Z1 expression promotes growth and tumor angiogenesis in breast cancer^44^ by increasing mRNA expression and producing VEGFA, a known factor highly expressed in breast cancers. Interestingly, *CYP4Z1* was found to be highly upregulated in DHSF-BR16 cells compared with MCF-7 cells.

We evaluated sensitivity/resistance of DHSF-BR16 cells to cytotoxic drugs used to pre-operatively treat the primary tumor from which the cell line was derived. In comparison with MCF-7 cell line, sensitive to the DNA damaging antibiotics such as anthracyclines^45^, DHSF-BR16 cells were, as mentioned above, more resistant to anthracyclines (although mainly to doxorubicin than to epirubicin), included in the neoadjuvant treatment administered to the patient. Several innate or acquired mechanisms of tumor resistance toward anthracyclines have been described mainly in in vitro studies. Some of them involve alterations in anthracycline metabolic enzymes: the main example is probably represented by *ABCB1* overexpression. Results showed that the higher levels of *ABCB1* mRNA, detected by RT-qPCR, observed in the DHSF-BR16 cell line compared with MCF-7 cells, could have contributed to the lower sensitivity of DHSF-BR16 cells to the tested anthracyclines.

A further example is represented by *AKR1B10*, encoding an aldose reductase involved in the reduction of biogenic and xenobiotic aldehydes and whose altered expression has been reported in doxorubicin resistant breast cancer cells^46^. Both DHSF-BR16 and MCF-7 cells were sensitive to taxanes being our cell model about threefold more sensitive than MCF-7. Interestingly, Rodrigues-Ferreira et al.^47^ reported that the aneuploidy significantly enhanced sensitivity to paclitaxel in human tumor cell lines. We could therefore hypothesize that the substantial taxanes sensitivity of DHSF-BR16 cells may be due to the high percentage of aneuploidy, nevertheless their reduced modal number of chromosomes rather than MCF-7 cells^10^.

Finally, *WNT5A*, a non-classical member of the WNT gene family, was highly upregulated in DSHF-BR16 cells. Prasad et al.^48^ reported that *WNT5A* is involved in several signaling pathways, among which the most important is related to breast cancer cell migration and invasion. Due to the high levels of *WNT5A* in DHSF-BR16 cell line, it may represent a relevant tumor model for an in-depth study of the WNT5A signaling.

Other genes involved in transcriptional regulation (i.e. *SOX2*, *YAP1* and *STAT3*), in angiogenesis (i.e. *VEGFA*) or in signal transduction (*CLDN3*) were found to be overexpressed in DHSF-BR16 cells compared with MCF-7 by RT-qPCR. These genes possess features that well contribute to the definition of specific characteristics of DHSF-BR16 cells. It has been recently demonstrated that *SOX2*, a transcription factor essential for the maintenance of proliferation and self-renewal of cancer stem cells, is related to breast cancer initiation^49^. *YAP1*, the core protein of the Hippo signaling pathway, has been associated with malignancy and immunosuppression. Its transcription has been recently reported to be enhanced by a novel lncRNA, RP11-323N12.5^50^. Furthermore, *YAP1* plays an important role in the active inhibition of Wnt/β-catenin signaling and is essential for downregulation of β-catenin nuclear activity^51^. *STAT3* has been suggested as a pro-survival factor in breast cancer and in several breast cancer cell lines^52^ and to promote tumorigenesis through the regulation of the expression of various target genes, including cell-cycle regulators, angiogenic factors and anti-apoptotic genes^53^. Some claudins, including *CLDN3*, are considered to play an important role in the EMT and to affect the chemosensitivity of invasive breast cancer cells^54^.

The described above genes possess molecular and functional characteristics of wide interest among researchers involved in the study of breast cancer biology. Of course, this cell model may offer the possibility of defining additional molecular mechanisms in which other differentially expressed genes compared with MCF-7 cell line, could be involved. This would increase the knowledge on this very heterogeneous disease, for instance by allowing the identification of new pharmacogenomic biomarkers.

In conclusion, in this work we prompted a cell line based on a quite rare event, that is the spontaneous immortalization. We reported a concise description of DHSF-BR16 cells that retained the phenotype characteristics of the primary tumor, and that are characterized by a high oxidative metabolism and a high stemness with the ability to form multicellular spheroids. Thus, the DHSF-BR16 cell line provides a good model to study the mechanisms responsible for the CSC quiescence/self-renew and to develop CSC-specific therapeutic targets, overcoming the tumor relapse.

## Supplementary Information

Supplementary Information 1.

Supplementary Information 2.
