# Supplementary material for: Establishment and characterization of a new spontaneously immortalized ER−/PR−/HER2+ human breast cancer cell line, DHSF-BR16
Source: Sci Rep. 2021 Apr 16;11:8340. doi: 10.1038/s41598-021-87362-0 (PMC8052418; doi:10.1038/s41598-021-87362-0)
Supplement: Supplementary file 1 — Supplementary Information 1. [file 41598_2021_87362_MOESM1_ESM.docx]

Supplementary Materials for

**Establishment and characterization of a new spontaneously immortalized ER^-^/PR^-^/HER2^+^ human breast cancer cell line, DHSF-BR16**

Stefania Nobili^1#^, Antonella Mannini^2#^, Astrid Parenti^1^, Chiara Raggi^2^, Andrea Lapucci^1^, Giovanna Chiorino^4^, Sara Paccosi^1^, Paola Di Gennaro^3^, Vania Vezzosi^5^, Paolo Romagnoli^2^, Tommaso Susini^1^, Marcella Coronnello^1*^.

**Supplementary Materials** include Supplementary Methods, Figures with Legends S1-S6, and Table S1. For Table S2 see the attached Excel file.

**Supplementary Methods**

**MCF-7 cell line and culture conditions**

MCF-7 cell line was obtained by ATCC, and maintained in Dulbecco’s Modified Eagle’s Medium (DMEM, Gibco Invitrogen) supplemented with 10% FBS (Gibco), 2 mM glutamine, and incubated at 37°C in humidified atmosphere with 5% CO_2_. Cells were weekly splitted at a subculture ratio of 1:5.

**Mycoplasma test**

The two cell lines were checked for the presence of mycoplasma with Venor GeM Mycoplasma Detection Kit, PCR-based (Minerva Biolabs).

**Evaluation of generation time and cell cycle progression**

Cell cycle progression and generation time has been evaluated in the same experiment. After removal of the monolayer culture medium, the cells were washed with phosphate buffer saline (PBS), magnesium and calcium ions free, then incubated with trypsin solution at 37°C until cell detachment. After trypsin inactivation with growth medium addition, cells were centrifuged, and the pellets were resuspended in the growth medium. The cell density was determined by counting the cells in a suspension sample by LUNA-II Automated Cell Counter (Logos-Biosystems). Cells were seeded at an initial density of 1x10^5^ cells/ml and subsequent changes in cell number were followed over 9 days. Growth curves were generated plotting the total cell division (TCD), as a function of time, calculated according to the formula:

**TCD =** $\frac{{log}_{10} \left( \frac{Nt}{N0} \right)}{{log}_{10} 2}$

in which N_0_ is the initial cell number (t_0_) and N_t_ is the cell number at time t.

To evaluate the cell cycle progression, daily fixed cells were stained with a PI staining technique, as described by Coronnello et al. (17). Briefly, aliquots of 1×10^6^ cells were washed twice in PBS (pH 7.4), centrifuged, fixed in 70% ice-cold ethanol, and stored overnight at 4° C in the dark. Cells were then rehydrated in PBS, and stained with PI solution (50 µg/ml) containing RNase A (5 U/ml) for 30 min. PI stained cells were acquired by FACSCanto and the red fluorescence emitted by PI was collected by a 620 nm long-pass filter, recorded as a measure of the amount of the bound-PI and distributed on linear scale.

For each histogram, the percentage of cells in the cycle phases (G_0_/G_1_, S and G_2_/M) was determined by a graphical curve fitting method of DNA distributions using FCS 6 Express software (De Novo Software).

**Expression of CD44/CD24 antigens.**

The expression of CD24 and CD44 surface antigens was measured by staining of DHSF-BR16 and MCF-7 cells with anti-CD24 (PE conjugate, clone SN3) and anti-CD44 (APC conjugate, clone IM7) antibodies, both from Life Technology. Briefly, after enzymatic detachment, cells were counted, resuspended with 1% Bovine Serum Albumin (BSA, Sigma) in PBS at a density of 0.5-1.0 x 10^6^/100µl and stained with the appropriate amount of the antibodies for 30 min at room temperature and at the dark. A negative cell sample for autofluorescence measurement is also included. After incubation, all samples were washed twice with 1%BSA in PBS, collected by centrifugation and suspended in 0.5ml of PBS for FACS analysis. The acquisition of the treated sample starts after finding the optimal reading conditions. Then the negative sample and the non-specific fluorescence sample are acquired, after which the reading of the treated samples begins. The CD24 and CD44 expression levels were reported as the percentage of positive cells in count versus fluorescence negative histograms.

**Immunocytochemistry**

Immunocytochemical staining, revealed either with fluorescent or enzymatic methods, was performed to detect nuclear receptor expression and cytokeratins in isolated cells.

Cells at 1x10^4^/ml density were centrifuged on glass slides in apposite cytofunnels (Shandon) at 5000 rpm for 5 min. The slides for the enzyme immunoassay were fixed in 10% formalin for 3-4 min and subsequently washed with PBS, dried in the air and labeled with anti-ER/PR and anti-HER2 antibodies; the slides for the fluorescence immunoassay were fixed in cold acetone (Sigma-Aldrich Inc.) for 6-7 min and stored at -20°C until labeling.

Cells were tested for the following list of monoclonal anti human primary antibodies (Abs): estrogen receptor (ER) (rabbit, clone SP1); progesterone receptor (PR) (rabbit, clone 1E2); HER2 (rabbit, clone 4B5); CK5/6 (mouse, clone D5/16B4), all from Ventana Medical System, Inc.. Other primary human antibodies tested are: CK8 (mouse, clone N1C1), CK18 (mouse, clone N2C2), EpCAM (mouse, clone N3C3) and E-Cadh all from GeneTex. The following secondary goat Abs were used: anti-mouse and anti-rabbit, either conjugated with Alexa Fluor (AF) 488 (green fluorescence) and AF594 (red fluorescence), all from Life Technology (Thermo Fisher Scientific Inc.). Abs dilutions were performed according to the manufacturer's instructions. Isotype-matched Abs were used as negative controls.

*Enzymatic immunocytochemistry.* To evaluate the persistence of the clinical phenotype obtained in 2015 on the tissue removed by the patient at the time of surgery, the same immunoenzymatic technique was performed on a cellular spot obtained with cells kept in culture for about 2 years. This technique was further supported by the immunofluorescent one. Enzymatic immunocytochemistry analyses were performed to examine exclusively ER, PR, and HER2 with an automated slide stainer and detection systems, according to manufacturer’s instructions (Ventana Medical System, Inc.), using secondary Abs conjugated with horseradish peroxidase (HRP) and DAB (3, 3-diaminobenzidine) chromogen (brown staining), followed by haematoxylin nuclear staining.

The samples were incubated for 15-30 min at 37°C. At the end of each incubation step, the slides were washed in the slide-stainer automatic VENTANA to stop the reaction and to remove the unbound material. In order to minimize the evaporation of aqueous reagents from the slide of the sample, Liquid Coverslip was also applied.

The results were compared with the breast ER^+^/PR^+^/HER2^-^ MCF-7 carcinoma line. Isotype-matched Abs were used as negative controls. The samples were observed under a light field microscope for a qualitative analysis. Adobe Photoshop CS2 software (Adobe Systems Incorporated, WA) was used for image processing and figure creating.

*Fluorescent immunocytochemistry*. Fluorescent immunocytochemistry analyses were performed on fixed cellular spots at room temperature (RT). Cells were hydrated with PBS and pre-treated first with 0.3% triton X-100 in PBS for 7 min, and then with 2% BSA in PBS for 45 min, for cell permeabilization and Abs unspecific binding blocking, respectively. Next, cells were incubated with the above-mentioned primary Abs for 90 min, revealed with the indicated secondary Abs for 60 min. Slides were mounted with ProLong Gold mountant with DAPI nuclear stain (Life Technology, blue fluorescence).

Slides were observed with Leica DMLB light microscope equipped for epifluorescence (Leica Microsystems GmbH). Images were acquired at 0.529 and 0.265 µm per pixel resolution, corresponding to 200x and 400x original magnifications, respectively, using Leica DFC200310 FX microscope digital color camera and LAS software with overlay module (Leica Microsystems GmbH). Adobe Photoshop CS2 software (Adobe Systems Incorporated, WA) was used for image processing and figure creating.

**Gelatin zymography**

To measure MMP2 and MMP9 activity, cells were seeded on collagen-coated wells. Following 48h stimulation, media were collected to clarify them by centrifugation and subjected to electrophoresis onto 8% SDS-PAGE containing 1mg/ml gelatin under non-denaturing conditions. Gels were washed with 2.5% Triton X-100 and incubated for 24 h at 37°C and 48h for MMP2 and MMP9, respectively, in 50mM Tris buffer containing 200mM NaCl and 20mM CaCl_2_, pH 7.4. Gels were then stained with 0.5% Coomassie brilliant blue R-250 in 10% acetic acid and 45% methanol and destained with 10% acetic acid and 45% methanol. Gelatinase activity was then evaluated by quantitative densitometry.

**Supplementary Figures**


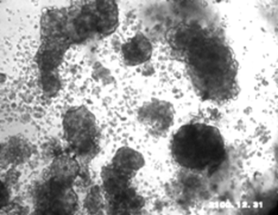

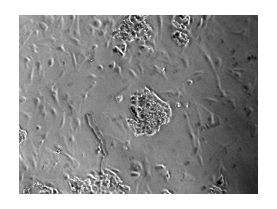

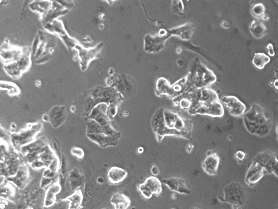

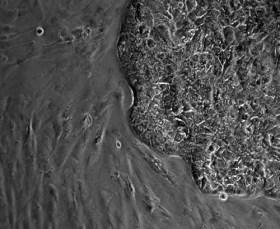

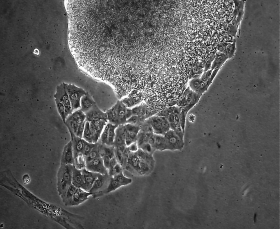

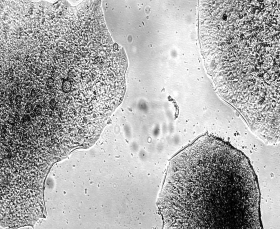


**a**

**b**

**c**

**d**

**e**

**f**

**Supplementary Figure S1**

**Isolation of DHSF-BR16 cells from invasive ductal breast carcinoma**. Series of images from organoids **(a)** up to established DHSF-BR16 cell line (**d,e,f**), passing from mixed cultures with fibroblasts (**b,c**). 100x magnification (a,b), 200x magnification (d), 320x magnification (c,e,f).


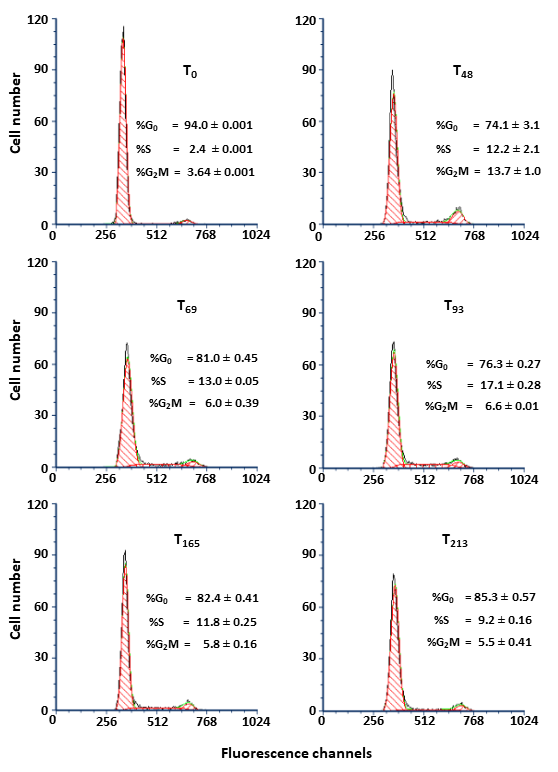


**A**

**B**

**Supplementary Figure S2**

**A, Cell cycle distribution of DHSF-BR16 gated cells** as a function of time. The percentage of cells in the various phases of the cell cycle is listed in each panel. On day 1, only 6% of cell population was in cycle, as evidenced by the high percentage of cells in G_0_G_1_ phase (94%); after 48 h, cells in S phase increased significantly to 12.2% as well as cells in G_2_M phase, that increased from 3.64% to 13.7%. Then, cells in S phase continued to increase up to 96 h and, subsequently, a progressive decrease in the percentage of cells in S phase and a progressive increase in the G_0_G_1_ phase was observed, while the percentage of G_2_M phase cells remained substantially constant.

**TCD**

**Time (h)**

**B, Growth curve of DHSF-BR16 cell line.** Starting with an inoculum of 1x10^5^ cells/mL, the cellular population increased exponentially for 48h, with a doubling time of 28h; thereafter, growth slowed down and it took other 5 days to get a new cell division; after 7 days, the total number of cell division (TCD) was approximately 3.

**
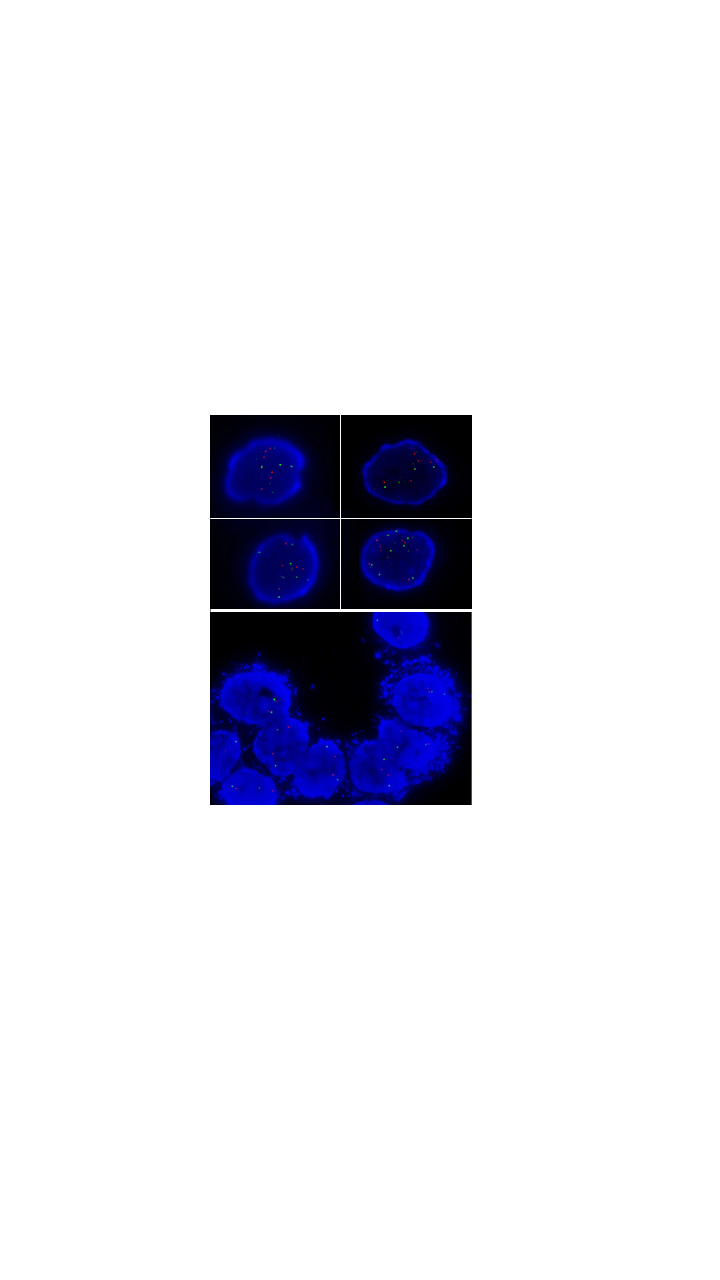
**

**DHSF-BR16 cells**

**MCF-7 cells**

**Supplementary Figure S3**

**FISH HER2-staining of DHSF-BR16 cells and MCF-7 cells (representative images).**

*HER2* gene amplification was demonstrated in DHSF-BR16 cells in contrast to the non-amplified MCF-7 cells (DAPI, blue fluorescence; HER2, red fluorescence; CEP17, green fluorescence).


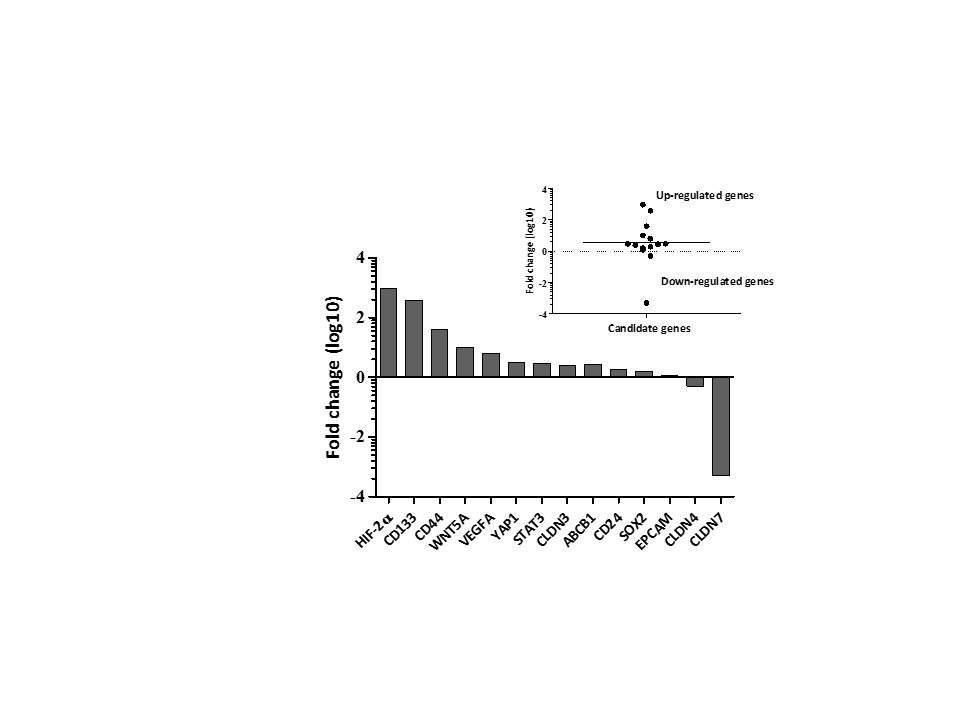


**Supplementary Figure S4**

**Graphed data from RT-qPCR of candidate genes (Table 1).**


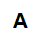

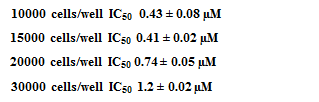


**% cell growth**

**[Doxorubicin, M]**


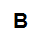


**TCD**

**cell number / well**

**Supplementary Figure S5**

**Representative cytotoxicity curves obtained with doxorubicin tested on DHSF-BR16 cells seeded at different densities (10.000 to 30000 cells/well). A,** Cells were exposed to doxorubicin for 72h and the percentage of cells was evaluated with the sulforhodamine test (see Materials and Methods Section).  In the range from 10,000 to 15,000 cells/well the IC_50_ difference was negligible. However, when densities were from 10,000 to 30,000 cells/well, the IC_50_ values a statistically significant increase, until to three folds, was observed (*p* <0.05). The IC_50_ values are the mean ± ES of at least three experiments conducted in quadruplicate.

**B**, untreated cells, and total cell divisions measured at T_72_h time; each point corresponding to cell number seeding at T_0_ h. At 72 h the highest TCD value was obtained at a 15,000 cells/well density.

**DHSF-BR16**

**MCF-7**


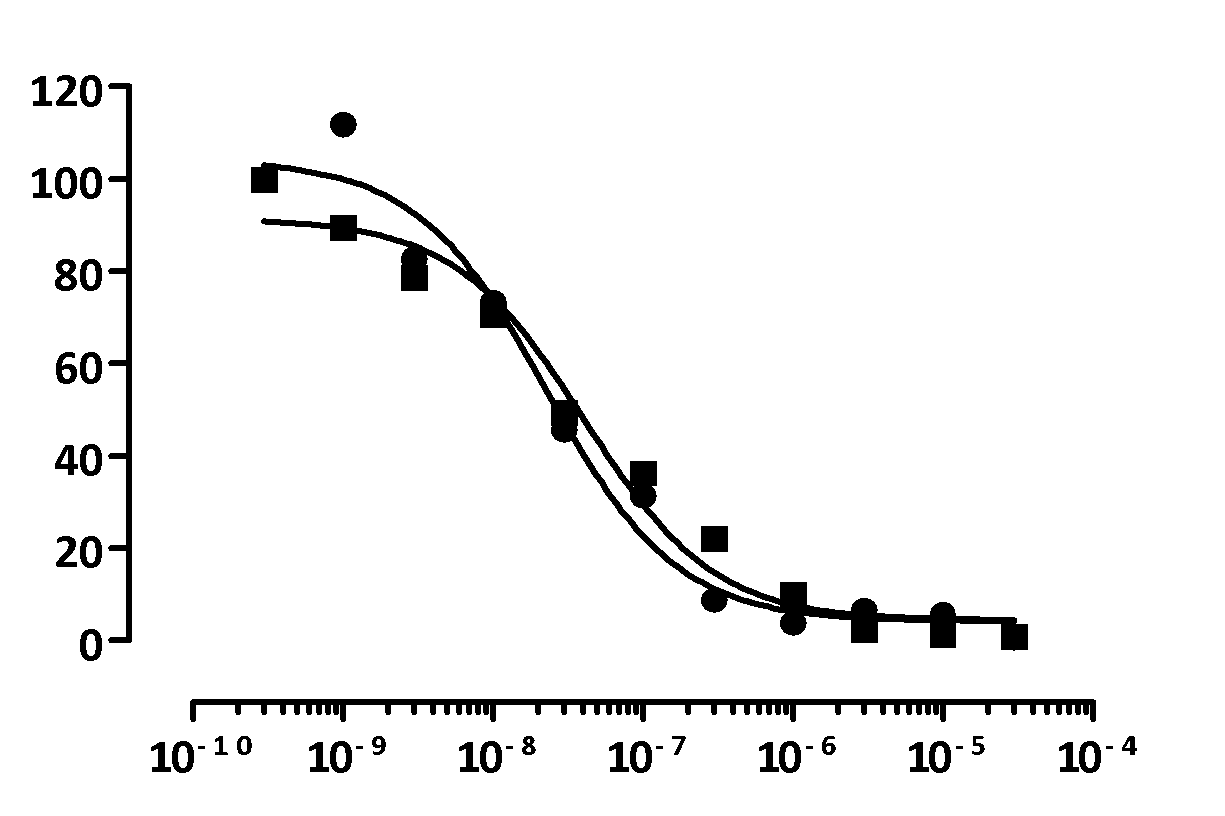


**% cell growth**

**% cell growth**

[**Epirubicin, M**]

[**Doxorubicin, M**]


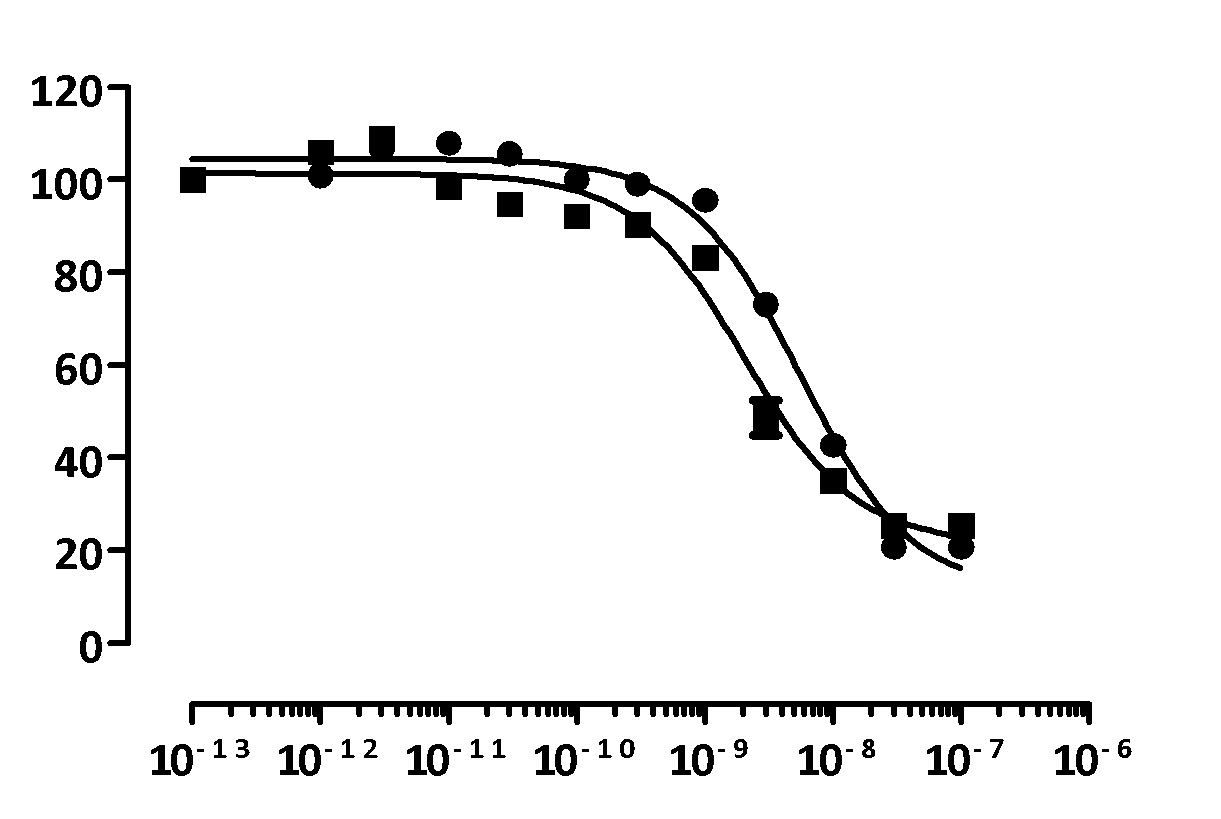

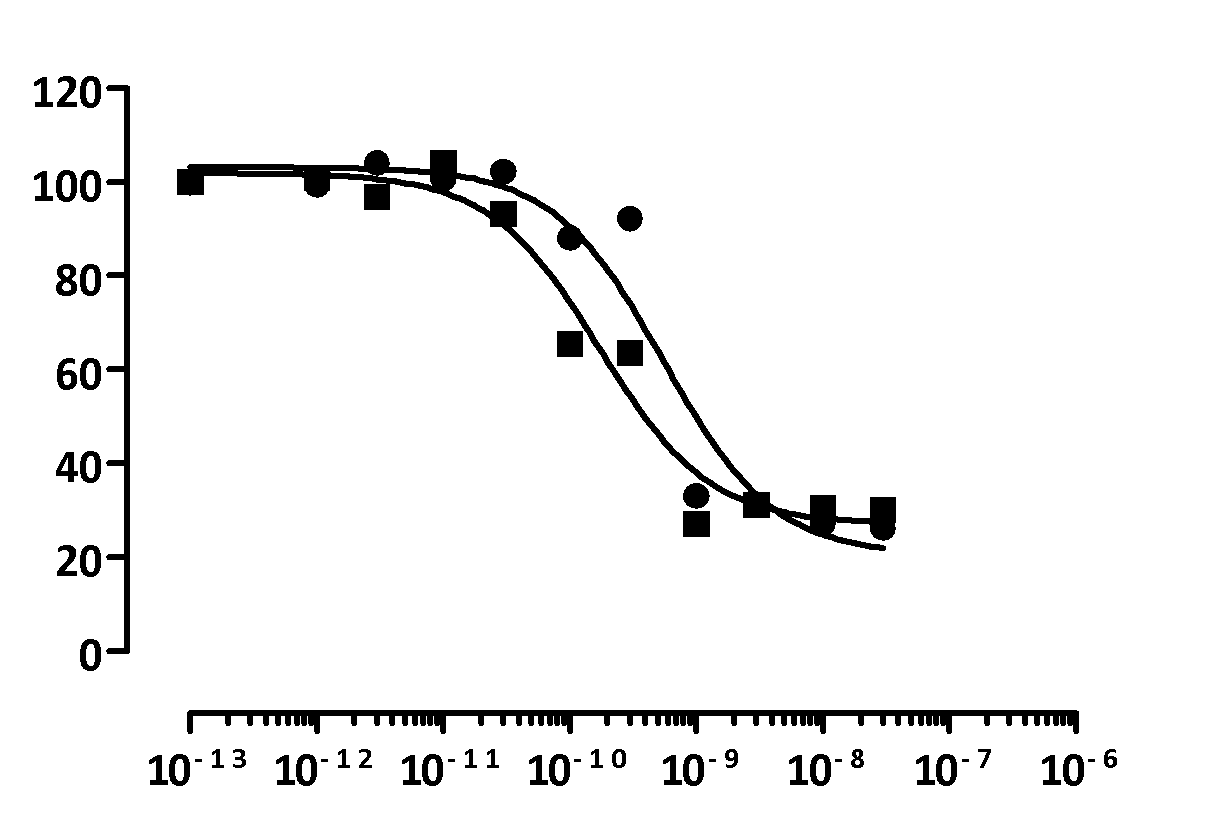


**% cell growth**

**% cell growth**

[**Docetaxel, M**]

[**Paclitaxel, M**]

**Supplementary Figure S6**

Representative cytotoxicity curves obtained with doxorubicin, epirubicin, docetaxel, and paclitaxel in the DHSF-BR16 breast cancer cell line compared to the MCF-7 cell line.

**Supplementary Tables**

**Supplementary Table S1**

**Index list of primer sequences used for RT-qPCR analysis.**

| Gene name | Forward sequence (5ʹ→3ʹ) | Reverse sequence (5ʹ→3ʹ) |
| --- | --- | --- |
| *h-TERT* | GACGTCTTCCTACGCTTCATGTGCC | CACCAACAAGAAATCATCCACCAAACGC |
| *CD44* | GTGATGGCACCCGCTATG | ACTGTCTTCGTCTGGGATGG |
| *VEGFA* | CACTGAGGAGTCCAACATCAC | AGGAAGCTCATCTCTCCTATGT |
| *CD24* | TAGGTACCACTATGGGCAGAGCAATGG | CCGGAATTCCGTTAAGAGTAGAGATGC |
| *CLDN3* | CTGCATGGACTGTGAAAC | AAAATATCAAGTGCCCCTTC |
| *CLDN4* | GAGCCATATAACTGCTCAAC | AGATAAAGCCAGTCCTGATG |
| *CLDN7* | CATAATTTTCATCGTGGCAG | ATACTCCTTGGAAGAGTTGG |
| *CD133* | GCTTCAGGAGTTTCATGTTGG | GGGGAATGCCTACATCTGG |
| *STAT3* | GGCATTCGGGAAGTATTGTCG | GGTAGGCGCCTCAGTCGTATC |
| *YAP1* | ACCCTCGTTTTGCCATGAAC | TTGTTTCAACCGCAGTCTCTC |
| *EPCAM* | TGTGGTGATAGCAGTTGTTGC | CTATGCATCTCACCCATCTCC |
| *SOX2* | ATGGGTTCGGTGGTCAAGT | GGAGGAAGAGGTAACCACAGG |
| *HIF-2α* | CGCTAGACTCCGAGAACAT | GGCTTGAACAGGGATTCAGT |
| *WNT5A* | GATGCCCTGAAGGAGAAGTACGACAG | TCCGACGTCTTGTTGCACAGGCG |
| *HER2* | GGGAAACCTGGAACTCACCT | CCCTGCACCTCCTGGATA |
| *ABCB1* | CAGCTATTCGAAGAGTGGGCACAAAC | GCCTCTGCATCAGCTGGACTGTTG |
| *BRCA1* | GACTGTTTATAGCTGTTGG | TTTTGGAAGTGTTTGCTACC |
| *BRCA2* | AATGTCAGACAAGCTCAAAG | TCATGTATTTTTCAGGTGGC |
| *NANOG* | GTCTCGTATTTGCTGCATCG | GAAACACTCGGTGAAATCAGG |
| *OCT4* | TTGTGCCAGGGTTTTTGG | ACTTCACCTTCCCTCCAACC |
| *BMI1* | TTGCTTTGGTCGAACTTGG | GTGCTTCTTTTGCAGACTGG |
| *KLF4* | AGACAGTCTGTTATGCACTGTGG | TGTTCTGCTTAAGGCATACTTGG |
| *C-MYC* | CGGAACTCTTGTGCGTAAGG | ACTCAGCCAAGGTTGTGAGG |
| *E-CADH* | AGGCCAAGCAGCAGTACATT | ATTCACATCCAGCACATCCA |
| *VIM* | ACACCCTGCAATCTTTCAGACA | GATTCCACTTTGCGTTCAAGGT |
| *ZEB1* | AAGAAAGTGTTACAGATGCAGCTG | CCCTGGTAACACTGTCTGGTC |
| *SNAIL* | CCTCCCTGTCAGATGAGGAC | CAAGGAATACCTCAGCCTGG |
| *LGR5* | CTTCCAACCTCAGCGTCTTC | TTTCCCGCAAGACGTAACTC |
| *CD13* | CAGTGACACGACGATTCTCC | CCTGTTTCCTCGTTGTCCTT |
| *Zeb2* | AGGGACAGATCAGCACCAAA | GTGCGAACTGTAGGAACCAG |
| *SLUG-2* | ACAGCGAACTGGACACACAT | GATGGGGCTGTATGCTCCT |
| *ABCG2* | GGCTTTCTACCTGCACGAAAACCAGTTGAG | ATGGCGTTGAGACCAG |
| *BMP7* | CAGCCTGCAAGATAGCCATT | AATCGGATCTCTTCCTGCTC |
| *LIN28A* | CAAAAGGAAAGAGCATGCAGAA | ATGATCTAGACCTCCAGAGTTGTAGC |
| *BMP4* | AGCGTAGCCCTAAGCATCAC | AGTCATTCCAGCCCACATCG |
| *CD90* | AGAGACTTGGATGAGGAG | CTGAGAATGCTGGAGATG |
| *NFKB1A* | CTCCGAGACTTTCGAGGAAATAC | GCCATTGAAGTTGGTAGCCTTCA |
| *NOTCH1* | GCAGTTGTGCTCCTGAAGAA | CGGGCGGCCAGAAAC |
| *HNF4* | CTCGTCGACATGGACATGGCCGACTAC | GGCTTGCTAGATAACTTCCTGCTTGGT |
| *GAPDH* | GATCATCAGCAATGCCTCCT | TGTGGTCATGAGTCCTCCCA |
| *18S* | CGGCTACCACATCCAAGGAA | GCTGGAATTACCGCGGCT |

**Supplementary Table S2 – Microarray expression profiling: bioinformatic and functional analysis (Excel file)**
